# Microbial control of arthropod-borne disease

**DOI:** 10.1590/0074-02760160373

**Published:** 2017-02

**Authors:** Miguel A Saldaña, Shivanand Hegde, Grant L Hughes

**Affiliations:** 1 University of Texas Medical Branch, Department of Microbiology and Immunology, Galveston, TX, USA; 2 University of Texas Medical Branch, Department of Pathology, Galveston, TX, USA; 3 University of Texas Medical Branch, Institute for Human Infections and Immunity, Galveston, TX, USA; 4 University of Texas Medical Branch, Center for Biodefense and Emerging Infectious Disease, Galveston, TX, USA; 5 University of Texas Medical Branch, Center for Tropical Diseases, Galveston, TX, USA

**Keywords:** microbiome, paratransgenesis, mosquito, arbovirus, vector control, malaria

## Abstract

Arthropods harbor a diverse array of microbes that profoundly influence many aspects of host biology, including vector competence. Additionally, symbionts can be engineered to produce molecules that inhibit pathogens. Due to their intimate association with the host, microbes have developed strategies that facilitate their transmission, either horizontally or vertically, to conspecifics. These attributes make microbes attractive agents for applied strategies to control arthropod-borne disease. Here we discuss the recent advances in microbial control approaches to reduce the burden of pathogens such as Zika, Dengue and Chikungunya viruses, and *Trypanosome* and *Plasmodium* parasites. We also highlight where further investigation is warranted.

Vector-borne diseases (VBD) are responsible for inordinate mortality, morbidity and economic loss worldwide. One of the most important groups of pathogen-transmitting vectors are the mosquitoes, including species within the *Anopheles*, *Aedes* and *Culex* genera. Particularly well studied are the *Anopheles* mosquitoes that vector *Plasmodium* parasites that cause malaria in humans. While five *Plasmodium* parasites cause malaria, *Plasmodium falciparum* is the major cause of this disease in sub-Saharan Africa (Snow et al. 2005). *Aedes* mosquitoes are notorious for vectoring arthropod-borne viruses (arboviruses) including flaviviruses such as Dengue virus (DENV), Yellow fever virus (YFV) and Zika virus (ZIKV), and also the Alphavirus, Chikungunya virus (CHIKV) (Bhatt et al. 2013, Weaver & Lecuit 2015, Weaver et al. 2016). *Culex* mosquitoes are known vectors of West Nile virus (WNV) and other encephalitic viruses, as well as filarial nematodes. Other than mosquitoes, *Phlebotominae* and *Simuliidae* flies are responsible for transmitting pathogens that cause Leishmaniasis, Onchocerciasis, as well as other neglected tropical diseases. In Africa, several species of tsetse flies vector *Trypanosomes* that cause sleeping sickness in humans and nagana in livestock. Further vectors include Triatomine bugs that transmit *Trypanosomes* that cause Chagas disease, which infects an estimated 6 million people in Latin America (Bern 2015). Ticks also transmit a variety of pathogens including viral, bacterial and protozoan parasites (Dantas-Torres et al. 2012). While traditional and contemporary control strategies have made great progress to control malaria and other neglected tropical diseases, the incidence of other diseases has been on the rise. Current disease prevention strategies often rely on vector control as effective vaccines are not available for many pathogens, however vector control strategies are becoming ineffective, mainly due to insecticide resistance emerging in many vectors (Naqqash et al. 2016, Ranson & Lissenden 2016). Taken together, novel strategies for control of VBD are urgently required. The current global ZIKV pandemic, and the reemergence of YFV in Africa and *Leishmania* in the Middle East stress this need for novel control tools against emerging and re-emerging pathogens (Al-Salem et al. 2016, Barrett 2016, Weaver et al. 2016). To this end, microbial-based intervention strategies are gaining considerable traction as a novel means to control VBD. In this review we highlight the recent advances in the use of symbionts to suppress pathogens in their vectors by drawing upon examples of viral, bacterial and fungal symbiosis in various vector species. Most studies have focused on mosquito vectors but where possible we include examples from other vector systems.

*The vector microbiome* - The advent of High Throughput Sequencing (HTS) technologies has expanded our understanding of the composition of the microbiome of many vector species. The microbiome is composed of viruses, bacteria, fungi and protozoa, however pathogens that vectors transmit can also be considered as constituents of the microbiome. Microbial association with the host can be facultative or obligate, and the nature of these host-microbe interactions, which range across a spectrum from parasitic to mutualistic, is likely fluid and depends on factors such as the host and environment (Casadevall et al. 2011). Microbes can have an intracellular or extracellular lifestyle, and possibly transition between both. Microbiota can also preferentially reside in specific host organs and tissue including the midgut (the lumen or gut epithelia), fat body, salivary glands, ovaries and testes (Sharma et al. 2014, Segata et al. 2016, Tchioffo et al. 2016). In several of these tissues, the microbe has the opportunity to directly interact with invading pathogens.

Our most comprehensive understanding of vector microbiomes is derived from mosquitoes. Studies utilising HTS have revealed that the microbiome is often dominated by relatively few taxa, can be highly variable, and that this variation is influenced by factors such as host life stage, host sex, the sampling technique, and the biotic and abiotic environment (Boissière et al. 2012, Osei-Poku et al. 2012, Coon et al. 2014, Gimonneau et al. 2014, Duguma et al. 2015, Buck et al. 2016, Segata et al. 2016). HTS techniques are currently most effective in examining the bacterial microbiome, and such work suggests mosquitoes have a microbiota comprised of bacteria within the phyla *Proteobacteria*, *Bacteriodetes* and *Actinobacteria*, encompassing taxa such as *Serratia*, *Pseudomonas*, *Aeromonas*, *Elizabethkingia*, *Enterobacter*, and *Acintobacter* (Boissière et al. 2012, Osei-Poku et al. 2012, Coon et al. 2014, Gimonneau et al. 2014, Hughes et al. 2014a, Duguma et al. 2015, Buck et al. 2016, David et al. 2016, Segata et al. 2016). Similar to mosquitoes, ticks have been found to have diverse and complex microbiomes, with the microbial composition influenced by life history traits and diet (Menchaca et al. 2013). The microbiome of lone star tick, *Amblyomma americanum*, is composed of the pathogens *Anoplasma* and *Ehrlichia* as well as other symbiotic bacteria within the phyla *Proteobacteria*, *Bacteroidetes* and *Firmicutes* (Jasinskas et al. 2007, Fryxell & DeBruyn 2016). Microbiome analysis of the Rocky mountain wood tick, *Dermacentor andersoni* identified four prominent genera of bacteria: *Rickettsia*, *Francisella*, *Arsenophonus* and *Acinetobacter* (Clayton et al. 2015). In tsetse flies, three vertically transmitted bacterial symbionts, *Wigglesworthia*, *Sodalis*, and *Wolbachia* are often present in the host, in addition to other environmentally acquired commensal bacteria (Wang et al. 2013b).

There are few studies investigating the fungal microbiome (mycobiome) of vector species. Most approaches that do explore the diversity of fungal microbes in insects exploit culture-based methods (Ignatova et al. 1996, Marti et al. 2006, Gusmão et al. 2010). A yeast strain, *Wickerhamomyces anomalus,* was found in both the midgut and reproductive system of the Asian malaria vector, *Anopheles stephensi* (Ricci et al. 2011), and six different fungal species have been found in the midgut of sandfly vectors (Akhoundi et al. 2012). However, recently, HTS was used to examine the mycobiome of *Aedes triseriatus* and *Aedes japonicus* (Muturi et al. 2016a). This study found twenty-one distinct fungal OTUs, 15 of which were shared between *Ae. triseriatus and Ae. japonicus* (Muturi et al. 2016a). The majority of fungal taxa in these *Aedes* species were from the *Ascomycota* phylum (Muturi et al. 2016a). Similarly, the *Ae. albopictus* mycobiome is dominated by fungi within the *Ascomycota* in addition to other taxa within phylum *Basidiomycota* (Muturi et al. 2016b). While the role of the mycobiome in regulating vector competence is poorly understood, it is likely that fungi and yeast can have a similar impact on pathogen transmission as bacteria, as fungi produce antimicrobial molecules and influence host immunity (Lemaitre et al. 1996, Martin et al. 2015, Wang et al. 2015, Angleró-Rodríguez et al. 2016). For instance, it was recently reported that *Penicillium chrysogenum* increases the intensity of *Plasmodium* infection in *Anopheles* mosquitoes by suppressing mosquito immunity (Angleró-Rodríguez et al. 2016).

Characterisation of the viral microbiome (virome) of disease vectors is now also gaining attention. Metagenomic sequencing of mosquitoes revealed the presence of several species of plant, animal and bacterial viruses in the mosquito virome (Ng et al. 2011, Chandler et al. 2015). Similar studies in ticks also identified several viral families, including previously unknown viruses (Tokarz et al. 2014, Xia et al. 2015, Sakamoto et al. 2016). The effect on the host of many of these viruses is yet to be elucidated. In contrast, we know that tsetse flies harbor a salivary gland hypertrophy virus (SGHV), which is a rod-shaped, enveloped DNA virus that is transmitted both horizontally and vertically, and can become pathogenic, causing hypertrophy of the salivary glands and reduced fecundity and lifespan (Wang et al. 2013b). Interestingly, it appears that there is an interaction of SGHV with microbial symbionts residing in the fly, as aposymbiotic flies have reduced viral loads (Boucias et al. 2013, Wang et al. 2013a).

Complex host-microbe interactions dictate microbiome and host homeostasis of arthropods. While the factors that shape the composition of the microbiome are still under investigation in most systems, it is clear that environmental conditions (Zouache et al. 2010, Wang et al. 2011, Minard et al. 2013), and host genetics (Kumar et al. 2010, Oliveira et al. 2011, Stathopoulos et al. 2014, Soares et al. 2015, Pang et al. 2016) are important. For instance, silencing of an antimicrobial peptide in *Triatoma infestans* elevated bacterial load in the midgut which subsequently reduces *Trypanosoma cruzi* parasites, indicating that host control of the microbiome can influence pathogen dynamics (Buarque et al. 2016). Bacterial genetics also appears to be an important determinant of gut colonisation (Maltz et al. 2012, Pei et al. 2015), however like much of the work examining bacterial genetic factors that influence persistence in the mammalian gut, this area of study is in its infancy in arthropods. While we have a limited understanding of the factors that regulate homeostasis in vectors, insights can be drawn from model insects where these processes have been examined in more detail (Buchon et al. 2013, Erkosar et al. 2013, Broderick 2016). In insects, microbial interactions are known to influence many diverse phenotypes and processes including host nutrition, reproduction, immunity, behavior, survival and evolution (Engel & Moran 2013, Lewis & Lizé 2015, Shropshire & Bordenstein 2016, v, van Tol & Dimopoulos 2016). In arthropod vectors, these phenotypes can have important implications for vectorial capacity. Additionally, members of the microbiome can themselves modulate vector competence for a variety of pathogens, either by direct interactions with the pathogen or indirectly mediated by the host (Dennison et al. 2014, Hegde et al. 2015). While the influence of the microbiome on vector competence is likely multifaceted and complex, interplay between the microbiota and host immunity is one process that can alter pathogen levels (Xi et al. 2008, Dong et al. 2009, Carissimo et al. 2015). Given these interactions, it is unsurprising that these interactions can also be reciprocated, whereby pathogen infection, which stimulates host immunity, can alter the microbiome (Xi et al. 2008, Ramirez et al. 2012, Zouache et al. 2012, Vieira et al. 2015, Zink et al. 2015, Muturi et al. 2016a). This highlights the intricate dynamism between the host and the microbiome, which in part, is shaped by host immunity. From an applied perspective, these microbe-mediated alterations in vector competence can be harnessed for novel microbial pathogen control strategies.

*Innate anti-pathogen activity of microbes* - *Wolbachia* - The most extensively developed microbial strategy to alter the vector competence of mosquitoes utilises *Wolbachia*. *Wolbachia* is a common bacterial endosymbiont that infects approximately 60% of insects (Hilgenboecker et al. 2008). It has been extensively studied for its ability to manipulate the reproduction of its host, which enables the bacterium to spread through insect populations (Werren et al. 2008). Cytoplasmic incompatibility (CI) is one of the most widespread reproductive mechanisms *Wolbachia* employs. CI occurs when an infected male mates with a female that is uninfected, or infected with an incompatible strain of *Wolbachia*. These crosses result in embryonic lethality and provide a fitness advantage to the infected female counterparts in the population, facilitating *Wolbachia’s* spread within insect populations (Werren et al. 2008). *Wolbachia*-mediated CI is being exploited as a population suppression tool termed incompatible insect technique (IIT) (reviewed in Bourtzis et al. 2014), and has been deployed to suppress *Aedes* mosquito populations (O’Connor et al. 2012). However, after it became evident that the antiviral properties of *Wolbachia,* which were first discovered in *Drosophila* (Hedges et al. 2008, Teixeira et al. 2008) also occurred in mosquitoes against a broad range of pathogens (Kambris et al. 2009, Moreira et al. 2009, Hughes et al. 2011b), the use of this bacteria for population replacement control strategies has been explored with vigor. The ability of the bacterium to confer pathogen interference, and to rapidly invade populations due to a high vertical transmission rate and the induction of CI, make *Wolbachia* an attractive agent for applied control.

*Wolbachia* can interfere with the development of diverse pathogens transmitted by mosquitoes. The anti-pathogen phenotype is particularly noticeable when a strain of *Wolbachia* is artificially transferred (transinfected) into a vector creating a novel strain-host combination (Hughes & Rasgon 2014). Most attention has focused on *Ae. aegypti*, which is generally thought to be naturally uninfected by *Wolbachia*, however, intriguingly, an infection was recently reported in mosquitoes collected in Florida, USA (Coon et al. 2016). Two strains of *Wolbachia* were found in these mosquitoes, which were phylogenetically related to the *w*AlbA and *w*AlbB strains in *Ae. albopictus* (Coon et al. 2016). Transinfected *Ae. aegypti* have reduced vector competence to several important arboviruses such as DENV (Moreira et al. 2009, Walker et al. 2011, Joubert et al. 2016), YFV (Hurk et al. 2012), CHIKV (Moreira et al. 2009, Aliota et al. 2016b) and ZIKV (Aliota et al. 2016a, Dutra et al. 2016). *Wolbachia* infected *Ae. aegypti* are also less competent vectors for filarial nematodes (Kambris et al. 2009) and *Plasmodium* parasites (Moreira et al. 2009). In addition to arbovirus control approaches in *Aedes* mosquitoes, *Wolbachia-*based strategies are also under investigation to inhibit Japanese encephalitis virus (JEV) vectored by *Culex tritaeniorhynchus* (Jeffries & Walker 2015).

Antiviral activity is also seen when novel strains are transinfected into *Ae. albopictus* (Blagrove et al. 2011), which is naturally infected with two strains of *Wolbachia*, *w*AlbA and *w*AlbB. Here, these resident strains were removed by antibiotic treatment before introduction of the novel *w*Mel strain from *Drosophila*. These *w*Mel-infected *Ae. albopictus* have decreased vector competence for DENV compared to an uninfected line and the naturally double infected mosquitoes (Blagrove et al. 2011). The effect of natural *Wolbachia* infections on pathogen dynamics is more difficult to assess, as uninfected individuals need to be identified, or the infection cleared with antibiotic treatment, for comparison. Antibiotic treatment can also have confounding effects such as altering the microbiome (Hughes et al. 2014a) or affecting mitochondria (Ballard & Melvin 2007). With these caveats in mind, native *Wolbachia* infections have been shown to reduce WNV in *Cx. quinquefasciatus* (Glaser & Meola 2010) and DENV and CHIKV in *Ae. albopictus* (Mousson et al. 2010, 2012), 2012), but it is important to note that these naturally infected mosquitoes are still competent vectors. Conversely, the native *Wolbachia* infection in *Culex pipiens* has been shown to exacerbate *Plasmodium* titer compared to their uninfected counterparts (Zélé et al. 2014), and *Wolbachia* also protects the vector against the deleterious fitness effects of the parasite, thus extending host lifespan, which has implications for pathogen transmission (Zélé et al. 2012).

The development of *Wolbachia* control strategies for human malaria appears more complex compared to arboviral pathogens. Aside from the propensity of *Wolbachia* to increase *Plasmodium* titer in some circumstances (Hughes et al. 2012, Baton et al. 2013, Murdock et al. 2014), which may be an artifact due to the method of infection or artificial nature of some tripartite combinations used in laboratory studies (reviewed in Hughes et al. 2014b), there are challenges with stably transinfecting *Anopheles* mosquitoes. To overcome these issues, transient infection was used to rapidly asses the effect of *Wolbachia* on *Plasmodium*, and this technique found that the *w*MelPop and *w*AlbB *Wolbachia* strains blocked *P. falciparum* (Hughes et al. 2011b). The *w*MelPop strain has also been shown to interfere with *Plasmodium berghei*, a murine malaria model (Kambris et al. 2010). In groundbreaking work from Bian et al. (2013) *An. stephensi* was stably infected with the *w*AlbB strain of *Wolbachia*. These novel infections induced CI in *An. stephensi* and substantially blocked *P. falciparum* (Bian et al. 2013), offering promise for the use of this bacterium in malaria control approaches. However, the infection also exerted a considerable fitness cost on the mosquito (Bian et al. 2013, Joshi et al. 2014), which would need to be overcome for *Wolbachia* to spread in field populations.

Recently, natural *Wolbachia* infections in some *Anopheles* populations have been discovered (Baldini et al. 2014, Buck et al. 2016, Shaw et al. 2016)*.* These studies, in addition to the transinfection of *An*. *stephensi* (Bian et al. 2013), have overturned the dogma that *Anopheles* mosquitoes were recalcitrant to *Wolbachia* infection and were naturally uninfected across their range. The native infections were shown to affect host fitness and reduce *Plasmodium* loads compared to uninfected conspecifics (Shaw et al. 2016). More work is required to determine if these natural infections can be exploited for *Plasmodium* control or if the resident strains would complicate the spread of more useful transinfected strains (Jeffries & Walker 2016). Similarly, the recently discovered natural infections in *Ae. aegypti* could have implications for implementation of *Wolbachia*-based strategies (Coon et al. 2016). Other bacterial symbionts that are known to manipulate insect reproduction (Duron et al. 2008), in a similar fashion to *Wolbachia* such as *Spiroplasma* (Terenius et al. 2008, Segata et al. 2016), and bacteria related to *Arsenophonus* (Briones et al. 2008) have been found in mosquitoes, but their effect on host reproduction and vector competence remains to be elucidated.

*Gut associated microbes* - Bacteria that reside predominately within the midgut of vectors can have profound anti-pathogenic effects that could be exploited in novel vector control strategies. Early studies examined the interaction between microbes and pathogens in *Anopheles*-*Plasmodium* and *Triatomine-Trypanosome* systems (Beier et al. 1994, Straif et al. 1998, Eichler & Schaub 2002). Today, most research in this area focuses on *Aedes* and *Anopheles* mosquitoes and the influence of the microbiome on arboviruses and *Plasmodium* parasites, respectively. Research that investigates the influence of gut microbes on pathogen dynamics is usually undertaken by perturbing the microbiome by antibiotic treatment or through administration of cultured bacteria to the vector. Alternative approaches included using antibodies raised against the microbiota to manipulate the microbiome, or rearing gnotobiotic lines (Noden et al. 2011, Coon et al. 2014). Antibiotic treatment has been shown to increase the titer of DENV in *Ae. aegypti*, JEV in *Culex bitaeniorhynchus*, *T. cruzi* in *Rhodnius prolixus* and *Plasmodium* in *Anopheles* mosquitoes (Mourya & Soman 1985 , Xi et al. 2008, Dong et al. 2009, Kumar et al. 2010, Rodrigues et al. 2010, Castro et al. 2012). These findings suggesting that the microbiota is antagonistic to invading pathogens. Re-infection of bacterial taxa into the vector enables the anti-pathogenic properties of specific microbes to be identified. Using this approach, isolates of *Enterobacter*, *Acinetobacter*, *Pantoea*, *Pseudomonas*, *Serratia* and *Elizabethkingia* have been shown to inhibit *Plasmodium* (Cirimotich et al. 2011, Bahia et al. 2014, Ramirez et al. 2014). The *Enterobacter* Esp_Z isolate was shown to produce reactive oxygen species (ROS) that inhibited the malaria parasite (Cirimotich et al. 2011), while other bacterial taxa may have distinct modes of action against *Plasmodium* (Bahia et al. 2014). Intriguingly, a specific strain of *Serratia* that has enhanced motility suppresses *Plasmodium* compared to a non-motile strain, providing insights into the mechanism behind the interference phenotype and highlighting the importance of bacterial inter-strain variation on vector competence (Bando et al. 2013). In other work, *Enterobacter*, *Proteus* and *Paenibacillus* species have been shown to inhibit La Crosse virus (LACV) and DENV (Joyce et al. 2011, Ramirez et al. 2012). Strikingly, a *Chromobacterium* isolate has both anti-*Plasmodium* and anti-viral properties, and reduces the survival of larvae and adult mosquitoes, possibly linked to the secretion of metabolites such as cyanide (Ramirez et al. 2014). Secreted molecules that have anti-pathogen and entomopathogenic activity could be harnessed for novel biotechnology applications. Such products could be used against the vector or the pathogens they transmit, or alternatively, exploited as novel pharmaceuticals for use in humans or livestock.

In addition to studies on arboviruses and malaria, bacterial microbes can alter pathogens in other vector species. *Serratia*, which is a dominant component of the gut microbiome of Triatomine bugs, appears to be an important determinant of *Trypanosome* infection (Azambuja et al. 2004, da Mota et al. 2012). The trypanocidal activity of *Serratia* could be related to prodigiosin production, which affects the mitochondrial activity of the parasite, and the ability of this bacterium to attach to the parasite (Castro et al. 2007, Genes et al. 2011). Studies in sandflies imply that microbes reduce *Leishmania* parasite load (Schlein et al. 1985) while tsetse flies cured of their symbionts were more susceptible to *Trypanosome* infection (Wang et al. 2009, Weiss et al. 2013). In ticks, both positive and negative interactions between symbionts and pathogens have been observed. *Rickettsia bellii* is negatively correlated with *Anaplasma marginale* infection, and reductions in a *Francisella* symbiont leads to a lower titer of the pathogenic *Francisella novicida* (Gall et al. 2016). Perturbing the microbiome of *Ixodes scapularis* altered the peritrophic matrix of the arachnid and subsequently led to a reduction in the spirochete, *Borrelia burgdorferi* (Narasimhan et al. 2014).

Pathogen enhancement mediated by microbes has also been documented in mosquitoes. Suppression of the midgut microbiota by antibiotic treatment in *Anopheles* mosquitoes decreased O’nyong nyong virus (ONNV) infections (Carissimo et al. 2015), indicating that constituents of the microbiota are required for pathogen infection. Re-infection of live, but not heat-killed bacteria, into antibiotic treated mosquitoes reverted viral titers to levels comparable to untreated controls (Carissimo et al. 2015). These effects are in contrast to what is observed with *Plasmodium* which increase in titer after antibiotic treatment of mosquitoes (Dong et al. 2009, Kumar et al. 2010, Rodrigues et al. 2010). A similar pathogen enhancement effect was seen in *Ae. aegypti* re-infected with *Serratia odorifera,* which increases both DENV and CHIKV infections (Apte-Deshpande et al. 2012, 2014). The ability of bacterial taxa to both enhance and suppress pathogens in insects suggests complex interplay between the host, the microbiome and the pathogen, dictates vector competence. Furthermore, specific vector-pathogen-microbe combinations may have unique outcomes, which means intervention strategies need to be scrutinised thoroughly before implementation.

While studies examining the role of the bacterial microbiome on arthropod biology are expanding and providing insights into alternative approaches to control arthropod-borne disease, we have a very limited knowledge on the role of the virome or mycobiome on vector biology and vector competence. The yeast *W. anomalus* produces a toxin that has in vitro antiplasmodial activity (Valzano et al. 2016). Studies investigating the entomopathogenic fungi *Beauveria bassiana* indicate this fungal pathogen suppresses DENV titer in *Ae. aegypti* through activation of the Toll and Jak-Stat immune pathways (Dong et al. 2012). This antiviral property further supports the use of this microbe for novel microbial biopesticide applications. Recently it has become evident that mosquitoes are naturally infected with insect-specific viruses (ISV). These viruses, which are phylogenetically diverse, infect mosquitoes but do not replicate within vertebrate cells (Blitvich & Firth 2015, Bolling et al. 2015, Vasilakis & Tesh 2015). Interestingly, it appears that ISV can suppress arboviruses in mosquitoes, likely due to a process known as superinfection exclusion (Newman et al. 2011, Bolling et al. 2012, Crockett et al. 2012, Kenney et al. 2014, Kuwata et al. 2015, Hall-Mendelin et al. 2016). Most studies have used in vitro systems and focused on insect-specific flaviviruses although an insect-specific alphavirus has been shown to alter Sindbis virus titer in vivo (Nasar et al. 2015). These findings have raised the possibility that fungi and ISV could be used in applied control strategies but before this can be achieved, a more thorough understanding of the biology of these microbes is required. Studies should focus on examining the ecological range and infection frequency of these microbes in natural mosquito populations, understanding the nature of their association with the host and other microbes, and investigate the mechanisms in which they are acquired and transmitted.

*Engineering microbes to convey anti-pathogen activity* - Microbes that reside within the gut of vectors can be engineered to secrete anti-pathogen molecules, an approach known as paratransgenesis. Paratransgenic studies were initially pioneered in Triatomine bugs for control of Chagas disease (Durvasula et al. 1997, Beard et al. 2002). Here, the symbiotic bacterium *Rhodococcus rhodnii* was genetically manipulated to express antimicrobial peptides that were antagonistic to *T. cruzi*, the parasitic protozoan that causes Chagas disease. Expression of cecropin A eliminated or reduced the number of *T. cruzi* within *R. prolixus* (Durvasula et al. 1997). Ingeniously, the copraphagic tendencies, or probing of fecal droplets, of the insect were exploited to deliver the transgenic symbiont to the vector. An artificial mimic of *R. prolixus* feces spiked with transgenic *R. rhodnii,* which was probed by nymphs, facilitated symbiont acquisition (Durvasula et al. 1997). In field trials, around half of the nymphs exposed to the mimic were infected throughout their development (Durvasula et al. 1999).

After these seminal studies, Beard et al. (2002) detailed the requirements for successful paratransgenic strategies. These include: that a symbiotic relationship occur between the microbe and the host; that the microbe be readily culturable and transformable; transformation should not alter the symbiotic relationship with the host, alter microbial fitness compared to wild type conspecifics or make the microbe pathogenic; that the effector gene product should be secreted to interact with the pathogen; and that there must be an efficient way to deliver the microbe into the vector population.

Paratransgenesis is also being explored in other vector species, particularly *Anopheles* mosquitoes for the control of malaria, using bacterial microbes as delivery vehicles. Earlier studies investigated engineering effector protein secretion systems from *Pantoea agglomerans*, which was isolated from *Anopheles* mosquitoes (Riehle et al. 2007, Bisi & Lampe 2011). Importantly, transgenic bacteria administered to mosquitoes in sugar meals were seen to rapidly proliferate following a blood meal and had minimal impact on life history traits of the mosquito (Wang et al. 2012). The secretion of several effector proteins antagonistic to *Plasmodium* using the HlyA secretion system from *P. agglomerans* was shown to significantly reduce the intensity of *P. falciparum* in the mosquito gut (Wang et al. 2012). The mode of action and the targets of the anti-*Plasmodium* effector molecules has been comprehensively reviewed (Wang & Jacobs-Lorena 2013). *Asaia* is another candidate for paratransgenic control of malaria. This bacterium is important for larval development of *Anopheles* mosquitoes, is genetically tractable, appears to be easily acquired by mosquitoes and is vertically inherited to progeny (Favia et al. 2007, Chouaia et al. 2012). Secretion of the effector proteins, Scorpine and the anti-Pbs21 scFv-Shiva1 toxin fusion protein, from *Asaia* reduced oocyst intensity of *P. berghei* in the midgut compared to control bacteria (Bongio & Lampe 2015). *Elizabethkingia* is another dominant member of the mosquito microbiome that is transstadially transmitted. This bacterium has been genetically altered and re-infected into *Anopheles* and *Aedes* mosquitoes (Chen et al. 2015a), however the use of this microbe in paratransgenic control approaches may need to be reconsidered since it is potentially a human pathogen (Frank et al. 2013) and given its natural resistance to several antibiotics. Genomic and further epidemiological analysis may clarify if strains present in mosquitoes are the source of infection in humans (Kukutla et al. 2014, Teo et al. 2015, Garay et al. 2016).

Paratransgenic approaches are also being developed for the control of *Trypanosomes* vectored by tsetse flies. The symbiont *Sodalis glossinidius* has been manipulated to release anti-trypanosome nanobodies (antigen-binding molecules) in the fly gut (de Vooght et al. 2012, 2014). Strategies have proposed to couple paratransgenic *Sodalis* with *Wolbachia*, and exploit *Wolbachia’s* CI-mediated drive to spread the transgenic symbiont through the population. Modeling suggests that if *Wolbachia*-induced mortality is low and the anti-trypanosome molecule is effective, the incidence of disease could be successfully reduced (Medlock et al. 2013). Preliminary experiments such as the identification and culturing of microbes have been accomplished for paratransgenesis strategies in *Phlebotomus argentipes* sand flies for control of *Leishmania* (Hillesland et al. 2008).

In comparison to bacterial paratransgenic approaches, there are few examples of the use of viral or fungal symbionts for paratransgenic control. While fungal paratransgenic studies are limited in medical vector species, approaches are also being investigated to control agricultural pathogens (Hughes et al. 2011a). The identification of culturable fungi and yeast associated with vectors provides candidate microbes for further investigation (Ricci et al. 2010, 2011, Martin et al. 2015, Steyn et al. 2015). In a subtle variation on the paratransgenic theme, the fungal insect pathogen *Metarhizium anisopliae* has been manipulated to express effector molecules to inhibit *Plasmodium* in *Anopheles* mosquitoes (Fang et al. 2011). Expression of the peptide SM1, a single chain antibody, or the antimicrobial toxin scorpine, significantly reduced sporozoites in the salivary gland. Impressively, the expression of 8 repeats of SM1 and scorpine as a fusion protein reduced *Plasmodium* intensity by 98% (Fang et al. 2011). *M. anisopliae* is an insect pathogen that infects mosquitoes through direct contact with the cuticle, which may enhance infection of the vector, but its pathogenic nature would likely mean that continual release of the microbe would be required.

Viral paratransgenesis research has mainly focused on Densoviruses. *Aedes* DNV (AeDNV), which can be pathogenic to the mosquito host (Ledermann et al. 2004), has been manipulated to express foreign genes (Afanasiev et al. 1999). Expression of a toxin from AeDNV increased the pathogenic effects of the virus compare to wild type virus in *Ae. albopictus* (Gu et al. 2010), offering promise for this strategy to be employed as a biopesticide. An *Anopheles gambiae* DNV (AgDNV) has been characterised and used as an expression platform (Ren et al. 2008, Suzuki et al. 2014). Unlike AeDNV, AgDNV is not pathogenic to the mosquito host and has minimal impact on mosquito survival (Ren et al. 2014). While DNVs can be used to express proteins in mosquitoes and the virus infects relevant organs in the insect to interfere with invading pathogens, there are some obstacles that need to be overcome before these viruses can be used in the field for paratransgenesis. DNVs have small genomes, which can limit the size of the inserted transgenes and they often require wild type virus for effective viral packaging. In an elegant approach, recombinant AeDNV were engineered to express microRNAs that target host genes or to sequester host miRNA using antisense miRNA sponges (Liu et al. 2016). This strategy overcomes some of the challenges associated with expressing larger genes from these viruses and enables the use of RNAi, rather than effector molecules, for vector control.

*Microbes expressing RNAi* - A promising alternative to paratransgenesis has emerged whereby microbes are engineered to deliver double stranded (dsRNA) to insects. RNAi is a powerful tool to manipulate transcription that has been used extensively to elucidate the function of many insect genes. In particular this technology has been extremely valuable in identifying mosquito pathways and genes that influence pathogen dynamics (Xi et al. 2008, Garver et al. 2009, Souza-Neto et al. 2009) and other aspects of insect biology useful for mosquito control (Isoe et al. 2011, Thailayil et al. 2011, Figueira-Mansur et al. 2013). The RNAi pathway is also a natural defense strategy used by insects to inhibit invading viral pathogens (Keene et al. 2004, Sánchez-Vargas et al. 2009), and therefore lends itself to development for applied pathogen control of arboviruses. This approach is very flexible in that potentially any gene in the vector could be manipulated. In addition, a vast array of interfering molecules can be delivered to the vector to manipulate gene expression, including short-hairpin RNAs (shRNA), long hairpin RNAs (lhRNA), artificial microRNAs (amiRNA) or miRNA sponges. Engineered microbes could deliver multiple RNAi molecules, allowing several synergistic intervention strategies to be undertaken simultaneously, reducing the risk of evolution of resistance to a particular intervention approach. Theory predicts that viruses will not have the potential to evolve to such combinatorial intervention approaches (Leonard & Schaffer 2005), and experimental evidence shows that polycistronic expression of multiple shRNA can effectively inhibit DENV (Xie et al. 2013).

Delivery of RNAi to insects has been achieved with viruses, bacteria and yeast. For approaches targeting vector species, most strategies target host genes that when silenced induce mortality. These approaches can be considered as a novel species-specific insecticide. Other approaches have targeted genes that are important for reproduction, thereby reducing the fecundity of the insect. The use of bacteria for RNAi delivery is more complicated since the RNase III enzyme of the bacterium can degrade double stranded RNA (dsRNA). For many years, RNase III mutants of *Escherichia coli* have been used for RNAi silencing in the nematode *Caenorhabditis elegans* (Timmons et al. 2001). Similar approaches with RNase III mutant *E. coli* are effective for RNAi delivery to *Ae. aegypti* mosquitoes and *R. prolixus* bugs (Whyard et al. 2015, Taracena et al. 2015), while a *R. rhodnii* RNase III mutant was used to express RNAi in *R. prolixus* (Whitten et al. 2016). In contrast, wild type *R. rhodnii* bacteria were used to deliver RNAi molecules to Reduviid bugs that reduced fecundity of the insect (Taracena et al. 2015). Similarly, fungi have been used to express RNAi targeting several essential host genes to kill agricultural pests (Chen et al. 2015b, Murphy et al. 2016). The use of bacterial or fungal microbes as RNAi delivery vehicles appear promising for vector control and the next challenges in this field will be to use this approach to interfere with pathogen development within a vector.

*Deployment strategies* - Regardless of the nature of the anti-pathogenic phenotype, be it innate or engineered, a strategy to disseminate the symbiont effectively through the vector population to have a meaningful effect on disease incidence is required. *Wolbachia*-based approaches have a clear advantage in this regard as the bacteria can manipulate host reproduction by CI to spread, often rapidly, through vector populations. For example, *Wolbachia* was established into *Ae. aegypti* populations in Cairns, Australia, by release of infected adults (Hoffmann et al. 2011). Subsequent analysis of the infection frequency in mosquito populations two years after the release found the bacteria was near fixation at the release sites (Hoffmann et al. 2014). Other strategies have been proposed for bacteria that do not manipulate host reproduction, and these may be self-perpetuating or require continual releases depending on the biology of the symbiont and host. As mentioned above, one elegant approach used in paratransgenic strategies of Triatomine bugs exploits the unique coprophagic probing tendencies of *R. prolixus* (Durvasula et al. 1997). For readily culturable microbes, it has been suggested that dissemination of the microbe into mosquito populations could be achieved by spiking larval pools or by baiting sugar feeders (Schlein & Müller 2015). For the former, the microbe would either need to be transstadially transmitted or the adult would need to imbibe the microbe soon after emergence from the pupal case. It appears that gut bacteria are cleared during metamorphosis between mosquito life stages (Moll et al. 2001), but transstadial transmission may occur when other tissues like the malpighian tubules act as a reservoir for reinfection (Chavshin et al. 2015). In semi-field cage experiments, both sugar feeding stations and release of infected males was shown to be an effective method to perpetuate *Asaia* through *Anopheles* generations (Mancini et al. 2016). *Asaia* can be horizontally acquired and vertically transmitted, both maternally and paternally, which could perpetuate the infection (Favia et al. 2007, Damiani et al. 2008). A better understanding of the vertical and horizontal transmission of microbes and factors that influence microbiome homeostasis and composition is required before we can develop effective strategies for microbial release.

*Future directions for microbial control of arthropod-borne disease* - Although there is a rich history of insect symbiosis research, many questions are yet to be resolved, particularly with regard to vector microbiomes. For successful utilisation of microbes for applied control approaches several areas need to be addressed. Translating promising strategies that demonstrate that microbes can modulate vector competence in the lab to natural populations is a priority. For this to be achieved studies assessing the diversity of vector-associated microbes across diverse ecological niches is required. A related future direction is to examine both the host-microbe and host-microbe-pathogen tripartite interactions under differing environmental conditions such as temperature, as this variable has been shown to influence vector immunity and pathogen dynamics (Murdock et al. 2012). While a particular control strategy may be successfully implemented under one set of environmental and ecological variables, this may not hold true where conditions differ.

Another important area of future research is in understanding the factors that influence how microbes are acquired, maintained, and transmitted by vectors. This knowledge is essential for developing effective methods to deploy symbionts into a population. Dissemination of a symbiont into a vector population may be hindered by microbial competition within the host. For example, *Wolbachia* and other bacterial microbes such as *Serratia* and *Asaia* are antagonistic to one other (Hughes et al. 2014a, Rossi et al. 2015, Zink et al. 2015). Additionally, re-introduction of bacterial microbes into mosquitoes via a sugar meal was more successful when the native microbiota were suppressed by antibiotics, suggesting bacterial interactions in the gut dictate microbial colonisation (Ramirez et al. 2014). Cross kingdom interactions between bacteria and fungi, both positive and negative, were seen *Aedes triseriatus* and *Aedes japonicus* (Muturi et al. 2016a). Microbial interactions have also be documented in tsetse flies and ticks (Boucias et al. 2013, Wang et al. 2013a, Fryxell & DeBruyn 2016). As such, the issue of compatibility between microbial strategies could arise. For example, a *Wolbachia* based approach may interfere with an ISV strategy, as ISVs have recently been shown to be suppressed by *Wolbachia* antiviral activity (Schnettler et al. 2016). Furthermore, paratransgenic approaches using *Asaia* or *Serratia* may not be compatible with *Wolbachia* applied approaches. While such an occurrence could be overcome by assessing the most suitable approach for a particular invention, strategies that perpetuate and drive through populations may expand geographically and therefore preclude the use of another technology elsewhere. Furthermore, the compatibility between microbial-based approaches and other contemporary and conventional vector control strategies should be investigated.

Another challenge with using microbes that possess native anti-pathogenic effects is determining the mechanism(s) by which they interfere with pathogens. Studies are providing insights into the mechanism(s) of pathogen interference of *Wolbachia* (Pan et al. 2011, Caragata et al. 2013, Zhang et al. 2014), gut microbes (Azambuja et al. 2004, Cirimotich et al. 2011, Ramirez et al. 2014), and fungi (Valzano et al. 2016, Angleró-Rodríguez et al. 2016), however a more comprehensive mechanistic understanding would facilitate attempts to forecast the long-term evolutionary response of the pathogen to the intervention and assist in determining the most effective deployment regime for a particular approach. While attempts have been made to predict these long-term interactions (Bull & Turelli 2013), there are still unknown factors in these systems which makes these evaluations difficult. In contrast to this, the mechanism by which paratransgenic approaches inhibit pathogens is known as the effector molecule or RNAi cassette is engineered into the microbe. However, this means that all paratransgenic approaches have the unavoidable consequence that the microbe is genetically altered in some fashion.

For the ultimate utility of paratransgenic approaches, society needs to be receptive to this technology. Demonstrating the widespread benefits of these approaches by completing thorough and transparent research will enable societies and governments to make an informed decision of the risks and benefits of these novel control strategies. Further to this, the adoption of novel approaches to limit horizontal transfer of the transgene or the use of microencapsulation to contain microbes from environmental exposure will further enhance the safety of this technology (Arora et al. 2015, Mandell et al. 2015, Rovner et al. 2015). The success of the *Wolbachia* strategy employed by the Eliminate Dengue Campaign can provide a blueprint for other microbial-based strategies to address ethical, social and logistical hurdles. In particular, this program has received wide-spread community acceptance that can be attributed to their comprehensive risk assessments and outstanding outreach and engagement efforts (McNaughton 2012, McNaughton & Duong 2014, Murray et al. 2016).

*Summary* - While conventional vector control strategies have reduced the burden of some VBD, novel strategies are required. Microbial-based strategies are gaining traction as an alternative means to control VBD, as microbes have several desirable properties for applied control strategies, particularly the ability to disseminate through vector populations. Coupling this with the propensity of symbiotic microbes to interfere with pathogen development in the host or by engineering microbes to modulate vector competence vectors, microbial strategies offer great promise for control of important VBDs.
